# P18 Value of pneumococcal PCR in respiratory samples for exclusion of pneumococcal pneumonia

**DOI:** 10.1093/jacamr/dlad066.022

**Published:** 2023-06-14

**Authors:** Bruno Van Herendael, Philippe Van Lint, Katrien Hoet, Philippe Willems, Sam Van Goethem

**Affiliations:** Medical Microbiology Laboratory, GZA Hospitals Antwerp, Belgium; Medical Microbiology Laboratory, GZA Hospitals Antwerp, Belgium; Medical Microbiology Laboratory, GZA Hospitals Antwerp, Belgium; Medical Microbiology Laboratory, GZA Hospitals Antwerp, Belgium; Medical Microbiology Laboratory, GZA Hospitals Antwerp, Belgium

## Abstract

**Background:**

*Streptococcus pneumoniae* is the main aetiological agent in bacterial pneumonia, making it an essential component of respiratory multiplex PCR panels. Despite its relevance, PCR test results are difficult to interpret due to frequent non-pathogenic colonization on the mucosal surface of the upper airways.

**Methods:**

A retrospective study was conducted for lytA-gene results as target for *S. pneumoniae* in hospitalized patients that were diagnosed with pneumonia and on which a respiratory multiplex PCR panel was performed. Patients were classified as probable, possible or unlikely of having a pneumococcal pneumonia.

**Results:**

No difference in semi-quantitative Ct-values could be observed between the different groups. Sensitivity of 71.4% and specificity of 89.6% was found, corresponding with a negative predictive value and positive predictive value of 97.6% and 34.2%, respectively, when considering probable versus possible/unlikely.

**Conclusions:**

We conclude that a negative qPCR for the lytA-gene is a good marker for the inability to culture *S. pneumoniae* in a respiratory sample and is possibly sufficient to exclude *S. pneumoniae* as a causative agent, due to which we can regard it as a relevant addition to a syndromic multiplex PCR panel.

**Figure 1.**
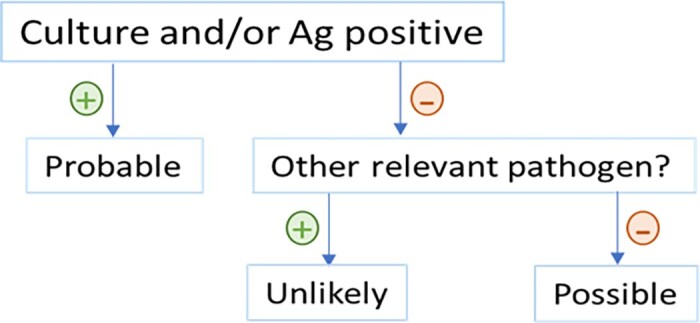
Probability of pneumococcal pneumonia categorization flow chart. Ag, pneumococcal urinary antigen test.

**Figure 2.**
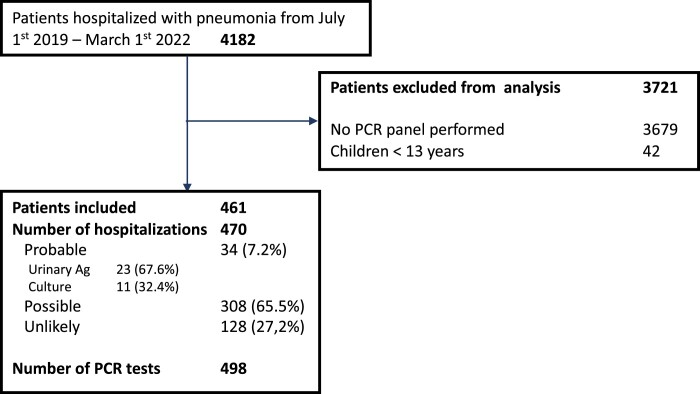
Inclusion flow chart and distribution among the probability of pneumococcal pneumonia categories. Urinary Ag, pneumococcal urinary antigen test.

**Figure 3.**
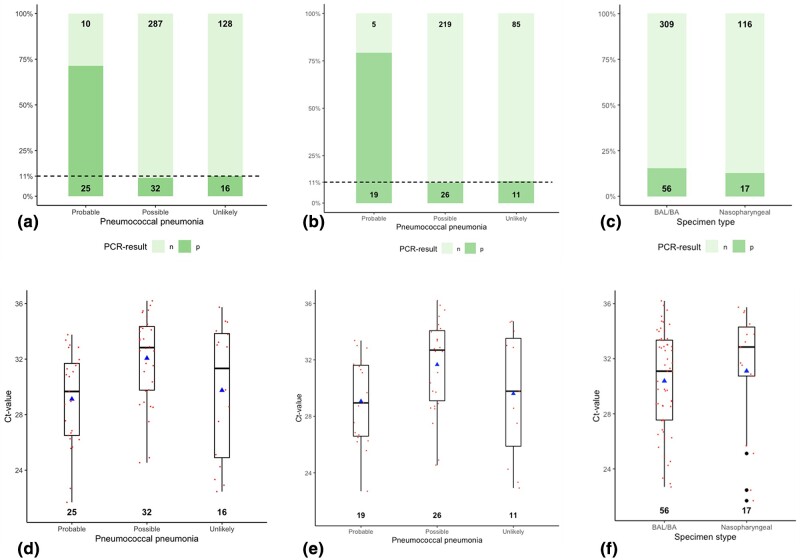
Results of the lytA-PCR. (a) Distribution of qualitative results according to the probability of having a pneumococcal pneumonia. (b) Distribution of qualitative results in deep specimen (BAL and BA) according to the probability of having a pneumococcal pneumonia. (c) Distribution of qualitative results according to the specimen type. (d) Distribution of semi-quantitative qPCR-results (Ct-value) according to the probability of having a pneumococcal pneumonia. (e) Distribution of semi-quantitative qPCR-results (Ct-value) in deep specimen (BAL and BA) according to the probability of having a pneumococcal pneumonia. (f) Distribution of semi-quantitative qPCR results (Ct-value) according to the specimen type. qPCR, quantitative polymerase chain reaction; BAL, bronchoalveolar lavage fluid; BA, bronchial aspirate; Ct, cycle threshold; n, negative; p, positive; blue triangle, Ct-value mean.

